# Dumpster diving for diatom plastid 16S rRNA genes

**DOI:** 10.7717/peerj.11576

**Published:** 2021-07-01

**Authors:** Krista L. Bonfantine, Stacey M. Trevathan-Tackett, Ty G. Matthews, Ana Neckovic, Han Ming Gan

**Affiliations:** 1 Centre for Integrative Ecology, School of Life and Environmental Sciences, Deakin University, Geelong, VIC, Australia; 2 School of Life and Environmental Sciences, Deakin University, Geelong, VIC, Australia; 3 GeneSEQ Sdn Bhd, Rawang, Selangor, Malaysia

**Keywords:** 16S rRNA, Metabarcoding, Algae, Biofilm, Bioindicator, Cyanobacteria, Bacillariophyta, Diatom

## Abstract

High throughput sequencing is improving the efficiency of monitoring diatoms, which inhabit and support aquatic ecosystems across the globe. In this study, we explored the potential of a standard V4 515F-806RB primer pair in recovering diatom plastid 16S rRNA sequences. We used PhytoREF to classify the 16S reads from our freshwater biofilm field sampling from three stream segments across two streams in south-eastern Australia and retrieved diatom community data from other, publicly deposited, Australian 16S amplicon datasets. When these diatom operational taxonomic units (OTUs) were traced using the default RDPII and NCBI databases, 68% were characterized as uncultured cyanobacteria. We analysed the 16S rRNA sequences from 72 stream biofilm samples, separated the chloroplast OTUs, and classified them using the PhytoREF database. After filtering the reads attributed to Bacillariophyta (relative abundance >1%), 71 diatom OTUs comprising more than 90% of the diatom reads in each stream biofilm sample were identified. Beta-diversity analyses demonstrated significantly different diatom assemblages and discrimination among river segments. To further test the approach, the diatom OTUs from our biofilm sampling were used as reference sequences to identify diatom reads from other Australian 16S rRNA datasets in the NCBI-SRA database. Across the three selected public datasets, 67 of our 71 diatom OTUs were detected in other Australian ecosystems. Our results show that diatom plastid 16S rRNA genes are readily amplified with existing 515F-806RB primer sets. Therefore, the volume of existing 16S rRNA amplicon datasets initially generated for microbial community profiling can also be used to detect, characterize, and map diatom distribution to inform phylogeny and ecological health assessments, and can be extended into a range of ecological and industrial applications. To our knowledge, this study represents the first attempt to classify freshwater samples using this approach and the first application of PhytoREF in Australia.

## Introduction

Diatoms are microscopic, unicellular powerhouses, supplying more than 40% of marine primary productivity worldwide and cycling oxygen, carbon, and silica through the world’s aquatic ecosystems (Mann, 1999). Sensitivity to environmental conditions, rapid growth rates, and durable silica frustules make diatoms robust indicators of current (Stevenson, Pan & Van Dam, 2010; Keck et al., 2016) and historic (Gasse et al., 1997) aquatic conditions. The taxonomic composition of diatom communities has been used to monitor changes in temperature (Descy & Mouvet, 1984), salinity (Gell, 1997), nutrient enrichment (Hall & Smol, 1992), pH (Ter Braak & Van Dame, 1989), and to assess pesticide (Larras et al., 2012) and pharmaceutical (Chonova et al., 2019) impacts on aquatic ecosystems. Accurate morphological identification presents a challenge for using diatom community patterns to assess ecological structure, function, and impacts. Significant expertise is required, and distinguishing morphological traits are often so subtle that trained taxonomists can reach different conclusions (Mann et al., 2010). Besides the subtlety, diatom morphology is also dynamic as they are ‘shape shifters’ whose size and morphological features vary with life stage and environmental conditions (Falasco & Badino, 2011; Medlin, 2018). Classification is then further complicated by the use of taxonomic references from well-studied regions, such as Europe and North America, to describe diatoms in areas with less diatom taxonomic research, such as Australia (Chessman et al., 2007; Hallegraeff et al., 2010).

Across the European Union, countries assess water quality using diatom-based indices, in accordance with the European Commission (2000). The algorithms that translate diatom counts to stream health scores require accurate diatom identification and quantification, as well as sufficient data to establish taxon-level ecological associations (Visco et al., 2015; Apothéloz-Perret-Gentil et al., 2017; Vasselon et al., 2017). However, misidentification is common, and limited underlying environmental data can produce different ecological values for the same species (Tapolczai et al., 2019). When the indices are applied outside of the region of the contributing data, they often perform poorly (Newall, Bate & Metzeling, 2006; Tan et al., 2017). Diatoms have rarely been used for bioassessment in Australia, but there is interest in developing regional indices (Chessman et al., 2007; Oeding & Taffs, 2017).

Given the array of important roles that diatoms play in aquatic ecosystems, the development of more efficient methods to identify and enumerate diatom communities could advance and expand stream health assessment (Visco et al., 2015; Pawlowski et al., 2016) and consequently, improve management and policy decision-making. Molecular techniques based on high-throughput sequencing (HTS) have provided new insights into diatom biology and ecology (Mann et al., 2010; Zimmermann, Jahn & Gemeinholzer, 2011), and ecologists are optimistic about the potential of diatom metabarcoding as a bioassessment tool. When tested, metabarcoding efforts have produced stream health scores similar to traditional approaches, but to-date, molecular diatom data are not part of routine biomonitoring programs (Kermarrec et al., 2013; Visco et al., 2015; Vasselon et al., 2017), perhaps because HTS techniques are generating as many questions as answers. Molecular data are challenging established phylogenetic relationships and uncovering cryptic diversity from samples examined under the microscope (Bennke et al., 2018; Medlin, 2018). Molecular data are also redrawing diatom distribution maps (Piredda et al., 2018). For example, *Skeletonema costatum* was once considered common and cosmopolitan, but molecular analysis exposed eight distinct species from previously identified *S. costatum*, some with specific, limited regional distributions (Sarno et al., 2005). However, the molecular signature of diatoms may not always reflect a biological species (Medlin, Williams & Sims, 1993) and it is not known if a single barcoding region effectively represents diversity across Bacillariophyta. Of the many gene regions that have been investigated for barcoding diatoms, the 18S nuclear rRNA (Zimmermann, Jahn & Gemeinholzer, 2011; Visco et al., 2015) and *rbcL* chloroplast markers (Evans, Wortley & Mann, 2007; Evans et al., 2008; Mann et al., 2010; Kermarrec et al., 2013) have been widely adopted (Rimet et al., 2016; Rimet et al., 2019) despite some limitations. For example, the highly conserved 18S nuclear rRNA gene region has failed to distinguish species within certain genera, such as *Skeletonema* and *Pseudo-nitzschia*, and some species may include several OTUs when using a 97% similarity cut-off (Piredda et al., 2018). Despite the uncertainty generated by reshuffling diatom phylogeny, over time, barcodes should help to resolve taxonomy in diatoms and other microalgae (Oliveira et al., 2018). A hidden resource for unravelling diatom mysteries may sit in the massive number of 16S rRNA sequences archived in public repositories. Since many bacterial 16S rRNA primers also have high affinity for eukaryotic plastid DNA, non-target sequences from chloroplasts and mitochondria are often co-amplified (Gan et al., 2019). These reads that are neglected in prokaryotic-based microbiome studies could provide a resource for identifying eukaryotic microalgae. The reads may also supply a more accurate measure of abundance than nuclear 18S rRNA, due to orders of magnitude less variability in gene copy numbers (Decelle et al., 2015; Needham & Fuhrman, 2016; Bennke et al., 2018). Rather than ignored, some plastid reads have also been misassigned as cyanobacteria in some databases due to the challenge in distinguishing chloroplast DNA from that of cyanobacterial ancestors (Bennke et al., 2018). If correctly attributed, the 16S rRNA marker offers the advantage of considering both cyanobacteria and photosynthetic eukaryotes using a single amplicon (Eiler et al., 2013; Lehmann et al., 2015; Bennke et al., 2018).

The potential for 16S rRNA sequences to describe the abundance and distribution of eukaryotic photoautotrophs can be explored now that a reference database exists for plastidal sequences. Decelle et al. (2015) produced the PhytoREF database by assembling all publicly available plastidal 16S rRNA sequences and amplicons resulting from Sanger sequencing of cultured microalgae (6,490 sequences). PhytoREF includes all major lineages of photosynthetic eukaryotes including three classes of diatoms: Coscinodiscophyceae, Fragilariophyceae and Bacillariophyceae and in silico analysis of the V5/V6 primer set has shown good database coverage (92%) of Bacillariophyta (Milici et al., 2016). PhytoRef focuses mainly on marine microalgae and so thus far, it has been used for classifying marine phytoplankton (Milici et al., 2016; Needham & Fuhrman, 2016; Bennke et al., 2018).

Taking advantage of these advances in reference databases, in this study, we present an approach for uncovering diatom assemblage data from 16S rRNA sequences. To our knowledge, the filtered and classification of diatom reads from a universal 16S primer has not been tested for freshwater communities, and PhytoREF has not been trialled in Australia. We developed this approach after failing to detect diatoms in the 23S reads (Sherwood & Presting, 2007) from our stream biofilm samples. We decided to probe the 16S rRNA sequences from the same biofilm samples to see if we could retrieve diatom community structure data from the chloroplast reads. We queried the chloroplast reads against the PhytoREF database to identify sequences belonging to diatoms and evaluated diatom community structure across different river segments. Then, in order to test the broader applicability of the method, we compared the diatom sequences from this study to three publicly available 16S rRNA sequencing datasets from Australia and demonstrated the capacity to mine digital diatom sequences. With further validation, this method could be applied to examine diatom phylogeny and improve biomonitoring at multiple spatial scales.

## Materials and Methods

### Field sampling

**Figure 1:**
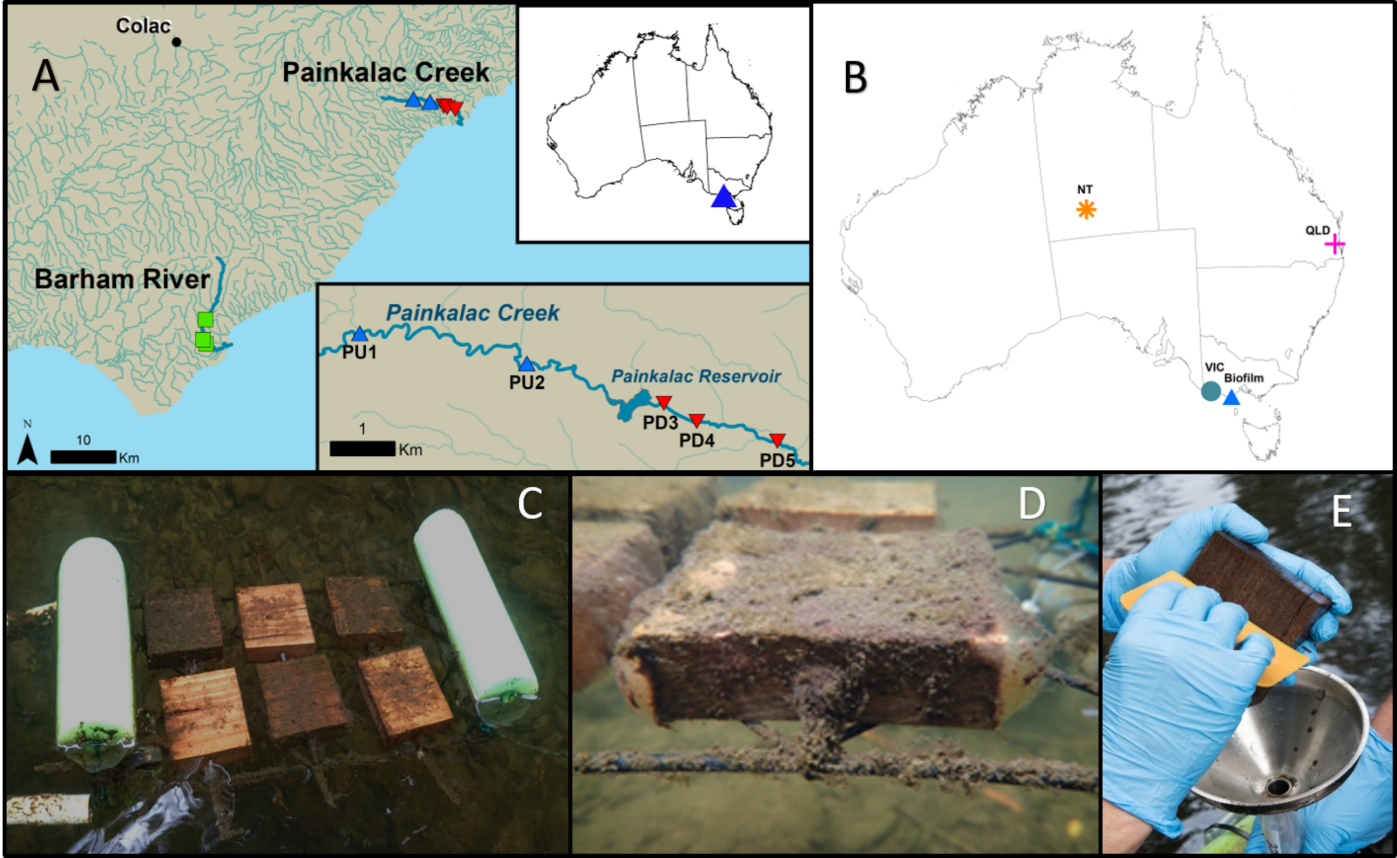
Stream biofilm sampling details and database sequence source locations. (A) The biofilm field sampling was conducted in Victoria, Australia. Two Painkalac Creek sites were upstream of Painkalac Reservoir (blue symbols) and three were downstream (red symbols). Three sampling sites were located on the Barham River (green symbols) (map source: Regional Surface Hydrology Lines. Geoscience Australia Crossman & Li2015) (B) Locations of samples from the stream biofilm sampling (Biofilm) and the publicly available 16S rRNA datasets (Kaestli et al., 2019) (NT); (O’Dea et al., 2019) (QLD); (Trevathan-Tackett et al., 2020) (VIC)). (C) Experimental floating frame with two sets of wood block substrate deployed two weeks apart. (D) Close-up view of a wood block coated in biofilm 21 days after deployment. (E) Scraping biofilm into stainless steel funnel inserted in Falcon©tube.

We collected biofilm samples from three stream segments in Victoria, Australia. One segment of the Barham River (B) was sampled along with two reaches in Painkalac Creek, one upstream (PU) and one downstream of the reservoir (PD) (Fig. 1A). Access was provided by the Victoria Department of Environment, Land, Water and Planning under permit # 10008062. We deployed wood blocks, composed of native mountain ash (*Eucalyptus regnans*), as a natural and consistent substrate for biofilm colonization (Ryder et al., 2006) on floating frames in eight locations (Fig. 1C). Two sets of three (60 cm^2^) blocks were anchored to each frame with overlapping but non-concurrent time periods (Fig. 1C). Between December 2018 and March 2019, nine sets of blocks were deployed at each of the eight sites to produce 72 biofilm samples. Only two sites were placed at PU because of limited summer water depth and access. Following each three-week growth period, which captures early and late biofilm successional patterns (Ryder et al., 2006), substrate blocks were removed and replaced and biofilms were scraped from the upper surface of the three blocks into a single composite sample (Figs. 1D, 1E). On two occasions, the sides of the blocks were collected and samples were examined and photographed using a compound light microscope for visual, qualitative documentation. A 0.33 mL subsample of the biofilm slurry was transferred to a one mL ZR BashingBead™Lysis tube (0.1 and 0.5 mm silica beads) with 0.66 mL of DNA/RNA Shield (Zymo Research, Irvine, CA), and vigorously mixed. A 0.5 mL subsample was transferred to a 47 mm Whatman GF/F glass fibre filter for subsequent lab analysis of chlorophyll concentration following standard protocol ( (American Public Health Association (APHA), 1995). All samples were stored at 4 °C until processing. Chlorophyll a concentrations per sample were calculated using the total sample volume in the Falcon^©^ tube and then converted to mass per unit area based on the combined surface area (180 cm^2^) of the three blocks (Biggs et al., 2000).

### Amplicon sequencing and bioinformatics

DNA from the preserved biofilm samples was extracted using a bead beating-based Zymobiomics Miniprep Kit (ZymoResearch) following the manufacturer’s instructions. Bead-beating was performed on a Vortex Genie2 at maximum vortex speed for 20 min. To improve DNA recovery, elution of DNA from the spin column used pre-heated TE buffer (56 °C) with an extended incubation time of 5 min. DNA concentration was quantified with a Qubit 4 Fluorometer (Thermo Fisher Scientific).

The purified DNA was sent to MR DNA (Shallowater, Texas, USA) for amplicon sequencing on the Illumina platform. Briefly, the V4 variable region of the 16S rRNA gene was amplified using the 515 (GTGYCAGCMGCCGCGGTAA) (Parada, Needham & Fuhrman, 2016)/806RB (GGACTACNVGGGTWTCTAAT) (Apprill et al., 2015) primer set with an in-line barcode on the forward primer. A single-step polymerase chain reaction (PCR) was performed using the HotStarTaq Plus Master Mix Kit (Qiagen, USA) under the following conditions: 94 °C for 3 min, followed by 30–35 cycles of 94 °C for 30 s, 53 °C for 40 s and 72 °C for 1 min, after which a final elongation step at 72 °C for 5 min was performed. Successful amplification and the relative intensity of bands were verified in 2% agarose gel. Multiple barcoded samples were pooled together in equal proportions based on molecular weight and DNA concentrations and then purified using calibrated Ampure XP beads (Beckman Coulter). The pooled and purified PCR product were sequenced on the Illumina MiSeq System (Illumina, San Diego, CA) using the run configuration of 2 × 300 bp.

Raw paired-end reads were processed using the MR DNA analysis pipeline. Briefly, paired-end reads were merged, depleted of barcodes followed by the removal of sequences shorter than 150 bp or with ambiguous bases. The sequence data from this study were deposited under BioProject PRJNA588337 in the Sequence Read Archive (SRA) of the National Center for Biotechnology Information (NCBI). Mothur was used for denoising, operational taxonomic unit (OTU) clustering at 97% similarity and chimera removal (Schloss et al., 2009).

The sequencing laboratory classified the 16S OTUs using BLASTn against a curated database derived from RDPII and NCBI (http://www.ncbi.nlm.nih.gov, http://rdp.cme.msu.edu) and we compared the results to those resulting from our tailored bioinformatics process. A subsequent classification step broadly separated the OTUs into four major groups (bacteria, archaea, mitochondria, chloroplast) using the QIIME2 q2-sample-classifier plugin (Bokulich et al., 2018) and its supplied Greengenes database (McDonald et al., 2012). The chloroplast-derived OTUs were further classified using the naive Bayes classifier in QIIME2, which has been trained using the PhytoREF database (Decelle et al., 2015). We herein refer to the nomenclature as assigned by PhytoREF which reflects shifting protist phylogeny and nomenclature (Adl et al., 2012; Adl et al., 2019; Guillou et al., 2012).

To determine whether chloroplast sequences accounted for the majority of 16S rRNA sequences (Eiler et al., 2013) and whether the proportion varied by location, we calculated the relative proportion of each of the four major groups (bacteria, archaea, mitochondria, chloroplast) across the total 16S sequence reads for each field site. We then compared the proportion of plastidal sequences (chloroplast + mitochondria) to prokaryotic sequences (bacteria + archaea). PhytoREF places diatoms within phylum Ochrophyta under the super-group Stramenopila (Decelle et al., 2015) and classifies Bacillariophyta as a class. We examined the proportion of Ocrophyta within the total chloroplast reads and the proportion of Bacillariophyta within the Ocrophyta reads. To compare the taxonomy assigned by PhytoREF (Decelle et al., 2015) to those returned by the NCBI non-redundant nucleotide database, we performed a similarity search (mega-blast) of the diatom OTUs identified with and without the ‘exclude environmental sample’ option selected.

### Method validation

To test the amplification of diatom chloroplast sequences by the bacterial primers, three in silico validation tests were performed. In the first test, the FastPCR in silico tool (Kalendar et al., 2017) was used to analyse the 515F/806RB primer set against the PhytoREF reference sequences (4641 sequences). For the second test, the FastPCR in silico tool (Kalendar et al., 2017) was used to analyse the primer set against 1747 publicly available, eukaryotic sequences from the NCBI nucleotide database obtained using the criteria ‘Bacillariophyta AND 16S’. For the third test, the primer alignment was investigated more closely by comparing a few randomly selected members of the phylum Ochrophtya to related phyla and to an *E.coli* sequence generated by the same primer set using ClustalX (Thompson, Gibson & Higgins, 2003).

To evaluate the performance of the PhytoREF database in classifying diatom chloroplast sequences deposited in the NCBI public database, a subset of 1666 Bacillariophyta sequences (complete and nearly complete genomes were excluded) were analysed in R 4.0.4 (R Core Team, 2019 using the DADA2 1.18.0 (Callahan et al., 2016) and Bioconductor 3.12 (Huber et al., 2015) packages. Taxonomy was assigned to the PhytoREF database (Decelle, 2015) through the *assignTaxonomy* function in the DADA2 package, using the Naive Bayesian Classifier method (Wang et al., 2007) with a 50% minimum bootstrap confidence threshold.

### Beta-diversity analysis of field biofilm samples

The read counts for each Bacillariophyta (diatom) OTU were used to construct a raw OTU table. Data were normalized to relative abundance (McKnight et al., 2019) and percent composition by OTU was calculated by applying a ‘standardize by total’ approach (Clarke & Gorley, 2015) based on the total Bacillariophyta read count for each sample. All multivariate analyses were performed using Primer v7 with PERMANOVA+ (Clarke & Gorley, 2015). The relative abundance values were square-root transformed to reduce the impact of a few dominant taxa in the Bray–Curtis similarity analysis (Bray & Curtis, 1957). The Bray–Curtis similarity matrix was conservatively constructed using diatom OTUs that had at least 1% abundance in a sample. An analysis using relative abundance is appropriate here based on consistent field and laboratory methods and orders of magnitude less variability in 16S gene copy numbers (Needham & Fuhrman, 2016; Bennke et al., 2018) than other molecular markers.

Differences between diatom communities among the river segments were examined using two-factor permutational multivariate analysis of variance (PERMANOVA) (Anderson, 2001) with 9,999 unique permutations. The random ‘site’ factor was nested within the fixed ‘segment’ factor. Homogeneity of dispersion between groups was tested using PERMDISP. A non-metric multidimensional scaling (nMDS) (Kruskal, 1964) plot was constructed to visualize the differences between communities, and pairwise SIMPER analysis (Clarke, 1993) was performed to identify the OTUs driving the significant differences among the sites. The five OTUs with the highest contribution to the dissimilarity plus a dissimilarity/SD ratio of greater than 1 (Clarke & Gorley, 2015) were selected to demonstrate differences between river segments, which were visualized using a heat map created in the ‘pheatmap’ package (Kolde, 2017) in R (v3.6.0; R Project for Statistical Computing, Vienna, Austria).

### Detection of selected diatom OTUs in other Australian 16S rRNA datasets

To test the capability of our approach for detecting diatoms in other microbiome datasets, we searched the NCBI-SRA database for publicly available 16S rRNA sequencing data from freshwater Australian studies. We were interested in the potential for the method to illustrate regional diatom distribution in Australia, so we selected three datasets that used a similar set of primers: two datasets from freshwater samples of a similar ecosystem to this study but from distant locations, and one dataset, from a brackish estuarine site of close proximity to this study (Fig. 1B, Table 1).

**Table 1:** Existing Australian 16S datasets used to compare the diatom reads in this study. Publicly available 16S rRNA gene datasets in NCBI-SRA database (Kaestli et al., 2019; O’Dea et al., 2019; Trevathan-Tackett et al., 2020).

**State**	**Sample type**	**Salinity description**	**Water regime**	**Sampling date**	**n**	**Reference**	**BioProject ID**
NT	biofilm water	freshwater	perennial ephemeral	June 2016	78	Kaestli et al. (2019)	PRJEB29669
QLD	water	freshwater	perennial	Mar/Apr 2018	13	O’Dea et al. (2019)	PRJNA484387
VIC	seagrass leaf	brackish	estuary flooded	July 2016	5	Trevathan-Tackett et al. (2020)	PRJEB36104

The diatom OTUs in our study were used as reference sequences to perform high-throughput sequence similarity searches using VSEARCH v.2.14.1 (Rognes et al., 2016) with minimum nucleotide identity cut-off of 97% (–usearch_global –id 0.97). This reference sequence based method streamlined the bioinformatics process and focused on the spatial distribution of the diatoms detected in our biofilm samples.

To compare the database diatom communities with each other and with our field data, all data were presence/absence transformed and a Jaccard similarity matrix was constructed (Legendre & Legendre, 1998) using Primer v7 with PERMANOVA+ (Clarke & Gorley, 2015). To visualize the community patterns across locations, the Bray-Curtis matrix was used to construct a shade plot. The distance among centroids was also calculated, and the resulting distance matrix was used to construct a non-metric multidimensional scaling (Kruskal, 1964) ordination.

## Results

### Taxonomic assignment of field biofilm diatom OTUs

After quality filtering, a total of 4.9 million reads were obtained from the 72 stream biofilm samples with an average of 68,273 (±5849) retained reads (Table S2). The smallest number of raw reads per sample was 34,222, with 30,175 passing the bioinformatics pipeline. Between 3.9 and 34.0% of the reads from each sampling location were classified as chloroplast sequences (mean = 16.5%; median = 15.0%; Fig. S1A, Table S2). Chloroplasts made up a higher proportion of the reads from the Barham River sites than from the Painkalac Creek sites. In 10 of the 72 samples, all from the Barham River, eukaryotic sequences (chloroplast + mitochondria) exceeded that of identified prokaryotic sequences (bacteria + archaea). Typical of samples rich in eukaryotic DNA (Parada, Needham & Fuhrman, 2016), the samples with a high proportion of chloroplast sequences also yielded 18S sequences, which were ignored as ‘unclassified’ in the Greengenes classification step.

Based on PhytoREF classification, 87.2% of the 1,464 chloroplast reads were assigned to Ochrophyta (Fig. S1B) of which, 63.1% were attributed to Bacillariophyta (Fig. S1C). Across the 72 biofilm samples, diatom (Bacillariophyta) sequences represented 36.4% of the total chloroplast reads, while green algae accounted for 10.4% (Streptophyta = 6.4%, Chlorophyta = 4%). The relative abundance of diatoms was proportionally calculated based on the 533 OTUs assigned as Bacillariophyta by PhytoREF (Decelle et al., 2015). After conservatively filtering for OTUs with at least 1% relative abundance in a sample, 71 OTUs were retained, herein referred to as ‘diatom OTUs’. These 71 diatom OTUs collectively and consistently made up more than 90% of the diatom reads in each stream biofilm sample. The percent identity of the 71 diatom OTUs assigned to the PhytoREF reference sequences ranged from 71.4% to 99.9% with a mean value of 87.6% (Table S3). Six of these OTUs were assigned to order (Surirellales, Naviculales or Chaetocentales) by PhytoREF, and three of these six OTUs could be classified down to genus (*Psammodictyon, Navicula* or *Chaetoceros*) (Table S1).

The taxonomic assignments for the 71 diatom OTUs were inconsistent using the two 16S amplicon databases. The bioinformatics pipeline based on the curated RDPII and NCBI databases assigned 48 of the 71 diatom OTUs (68%) as cyanobacteria. When we searched the stream biofilm sequences against the NCBI non-redundant nucleotide database, using the default BlastN search setting and database parameter, the majority of the top hits were returned as ‘uncultured bacterium’. When environmental samples were excluded from the search query, most of the OTUs were classified as chloroplast sequences, and discordance between NCBI and PhytoREF occurred mostly at the lower taxonomic rank. One major exception was for OTU6 that was classified by PhytoREF as *Chaetoceros,* a diatom genus, while the top NCBI hit was *Gomphoneis minuta* (99% sequence identity), a euglenid from a different phylum.

**Figure 2:**
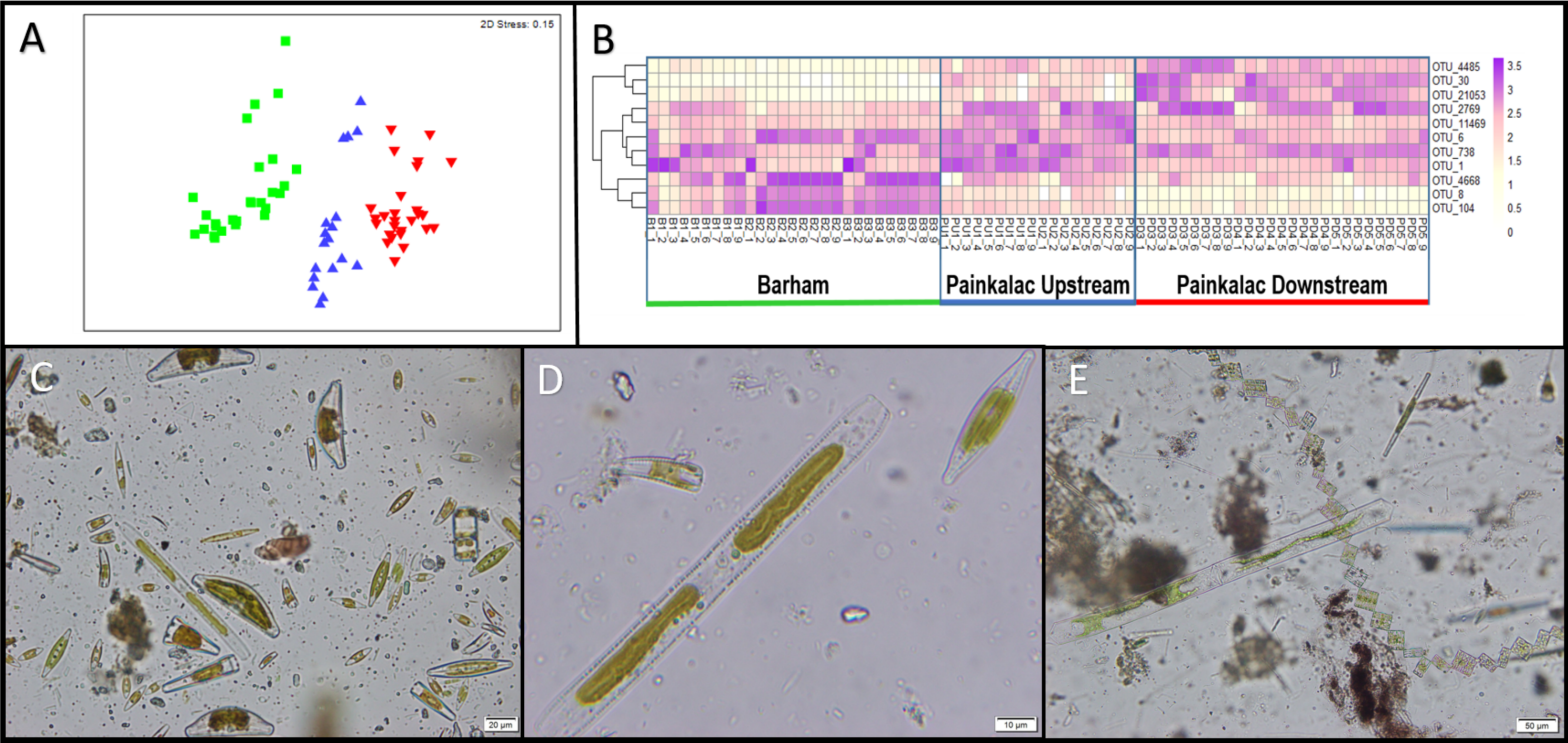
Diatom communities in stream biofilm samples. (A) nMDS ordination of Bray-Curtis distances of square-root transformed relative diatom abundance from 72 biofilm samples across 8 sampling sites within three river segments (colors; B = green, PU = blue, PD = red). (B) Heatmap showing relative abundance for 11 non-redundant OTUs identified by pairwise SIMPER analysis for 72 biofilm samples. (C) Sample B1_9 observed under microscope at 400×magnification. (D) A close-up of three diatoms from sample B1_9 at 1000×magnification. (E) Diatoms from sample PD5_6 observed at 200×magnification.

Visual patterns of diatom abundance, observed under the microscope, were consistent with the molecular patterns of total diatom abundance. Diatoms were most abundant in the Barham River (mean read abundance = 19.64), followed by the Painkalac downstream segment (mean read abundance = 7.75), and scant diatom representation in the upstream Painkalac segment (mean read abundance = 1.17). Of the three genera assigned by PhytoREF, only *Navicula* was visually observed in the samples. Four diatom genera that were not identified by their molecular signature (*Nitzschia, Gomphonema, Cymbella,* and *Melosira)* were observed within a single sample from the Barham River (Figs. 2C, 2D).

The general pattern of diatom read abundance is also consistent with the chlorophyll concentrations measured within each segment. Barham River biofilm samples had a mean chlorophyll concentration of 18.35 mg/m^2^ versus 7.60 mg/m^2^ for Painkalac downstream and 0.91 mg/m^2^ for Painkalac upstream samples.

### Method validation

According to the FastPCR in silico test with one mismatch allowed on the 3′-end, the primer set would amplify 90.8% of the PhytoREF sequences and 57.5% of the publicly available eukaryotic sequences labelled as ‘Bacillariophyta’ in the NCBI database. The Clustal nucleotide alignment confirmed the differences between the eukaryotic chloroplast sequences and prokaryote sequences (Fig. S1D). The Ochrophyta, and other eukaryote reads, showed high sequence conservation with no 3′ mismatches in the last 5 bases of both forward and reverse 16S v4-515F and V4-806RB primers. Two mismatches to the *E.coli* 16S rRNA (GT vs TA) were observed across all aligned non-*E.coli* 16S RNA sequences 15 bases upstream of the V4-806 primer-binding site (Fig. S1D).

In terms of database coverage, of the 1666 sequences classified as Bacillariophyta in NCBI, 660 were attributed to Bacillariophyta by PhytoREF. The sequences were distributed across 21 orders but only 33.7% of sequences were assigned at the family level and 22.4% were assigned a genus (Fig. S2).

### Distinct diatom assemblage in field biofilm samples

There were significant differences in diatom assemblage structure among the three river segments, indicated by the distinct clusters on the multidimensional scaling plot (Fig. 2A). The PERMANOVA results show that the composition of the diatom assemblage varied within (pseudo- *F*_5,71_ = 4.68; *P* < 0.001) and between the river segments (pseudo- *F*_2,71_ = 8.81; *P* = 0.003). There were no significant differences in the homogeneity of dispersion among sites (PERMDISP *P* = 0.74) or segments (PERMDISP *P* = 0.82). In the SIMPER analysis, there was redundancy in the 15 OTUs that contributed most strongly to the separation between river segments, which resulted in 11 non-redundant distinguishing OTUs (Fig. 2B, Table S1). OTUs 4668, 8, and 104 were more abundant in the Barham River, while OTUs 4485, 30 and 21,053 were characteristic of the Painkalac downstream sites. The Painkalac upstream samples shared OTUs with the other two reaches, but the patterns of abundance were different. For example, OTU 1 was more consistently abundant (min = 3.2%, max = 18.6%) upstream than in the other two reaches (PD: min 0.7%, max 14.9%; B: min = 0.3%, max = 43.6%).

### Occurrence of selected diatom OTUs in other Australian 16S rRNA datasets

**Figure 3:**
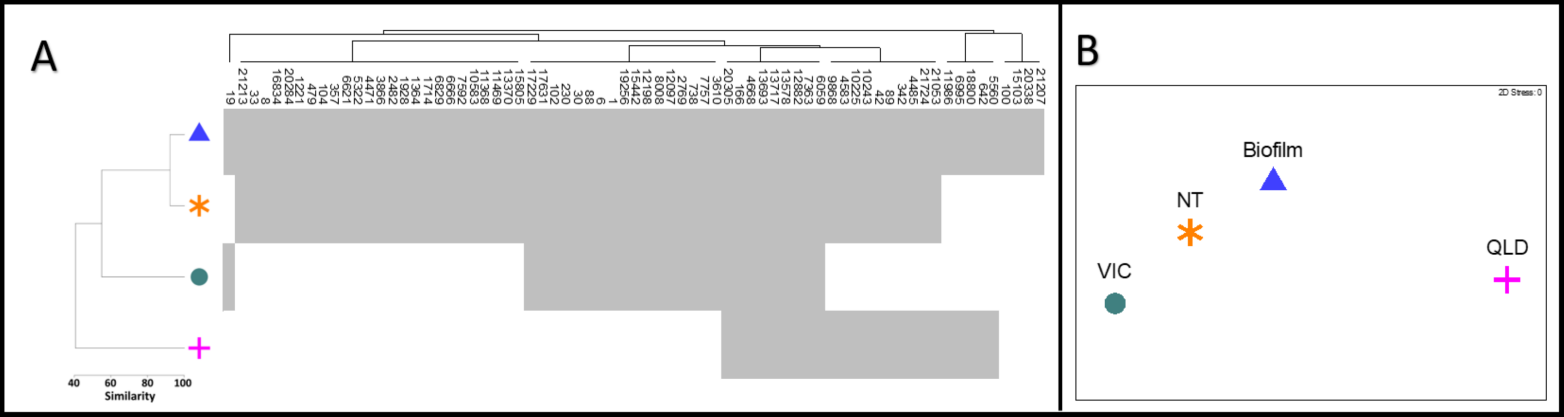
Diatom communities in stream biofilm and public database. (A) Shade plot depicting presence/absence of 71 diatom OTUs within each dataset. (B) nMDS based on presence/absence Jaccard similarity matrix including field data from this study (BIOFILM) and publicly available datasets (Kaestli et al., 2019) (NT); (O’Dea et al., 2019) (QLD); (Trevathan-Tackett et al., 2020) (VIC)).

Of the 71 diatom OTUs identified in this study, 67 were also detected in at least one of the other three Australian environmental 16S rRNA test datasets selected from NCBI-SRA (Table 1, Fig. 3A). Ten OTUs were observed across all four datasets, spanning freshwater and brackish habitats from far inland to estuarine ecosystems. Our stream biofilm samples shared the highest number of diatom OTUs (61) with the water and biofilm samples from the Northern Territory (NT) (Kaestli et al., 2019) (Fig. 3A). We detected diatoms in all of the NT samples, with diatom reads contributing a minimum of 0.2% of the total reads in a water sample and a maximum of 22.6% in a biofilm sample. The Victorian estuary site (VIC) was much closer geographically to our stream biofilm field locations, but only 27 of the diatoms were shared between the two sites (Figs. 3A, 3B). The lowest number of diatoms (24) and lowest similarity was shared with the freshwater samples from Queensland (O’Dea et al., 2019).

## Discussion

In this study, we show for the first time that the interrogation of 16S rRNA amplicon reads assigned to ‘chloroplast’ can provide insights into aquatic Australian diatom community composition and distribution. Additionally, this study showed that the PhytoREF database, typically used for describing marine phototroph lineages (Milici et al., 2016; Bennke et al., 2018), can provide some limited information for freshwater samples. The chloroplast reads that were filtered from the stream biofilm 16S amplicon data, and classified as diatom OTUs, showed distinct assemblage patterns at the river segment level. Further, OTUs shared between our field samples and public database samples described diatom occurrence across habitats at a continental scale.

We have shown that useful diatom community data can be constructed from mined 16S sequences but our experiment was not designed with this research in mind. Rather, we developed this approach in an attempt to characterise a community that was absent from our molecular data. Our biofilm field experiment employed a multi-metabarcoding approach, which included the 23S amplicon to detect photosynthetic eukaryotes (Sherwood & Presting, 2007), but no diatoms were detected. Using the method we present here, we uncovered the presence of a hidden resource within our 16S sequences but the quality of that resource remains uncertain based on limited reference database quality and knowledge gaps around non-target primer performance.

In examining the prokaryotic community structure based upon our 16S data, more sequences were discarded than used in 10 of our 72 samples. This highlights that non-target reads are not only a waste of sequencing power, but also suggests a missed opportunity for characterizing photosynthetic eukaryotes. We also identified misclassifications of diatoms as cyanobacteria, which could falsely inflate estimates of cyanobacterial abundance using the amplicon approach (Bennke et al., 2018) and thereby affect ecological inferences. Misclassifications are particularly relevant if cyanobacteria are to be used as bioindicators, as suggested by Mateo et al. (2015).

### Method validation

Research on the performance of prokaryotic primers in amplifying eukaryotic plastids has focused on reducing non-target amplification so little is known about primer bias and taxonomic coverage in non-target taxa. While our study does not comprehensively investigate these issues, our in silico results provide some relevant details. The primer set 515F/806RB amplified 90.8% of the PhytoREF sequences and about half (57.5%) of the publicly available ‘Bacillariophyta’ sequences deposited in NCBI. The NCBI sequences were mostly partial (95%), many lacked the target region, and some may be incorrectly annotated in the database. It is difficult to assess primer bias across taxa when 61% of the NCBI sequences (1017) are from ‘uncultured diatoms’ and only 39.6% of the sequences were attributed to Bacillariophyta when analysed against PhytoREF. The plastid sequences in NCBI have largely been discarded as nuisance reads so the nature and quality of these ‘trashed’ sequences requires more investigation.

Based on our alignment, there were no 3′ mismatches in the last five bases of either primer (Fig. S1D) which are generally thought to prevent amplification (Sipos et al., 2007; Hanshew et al., 2013). Because chloroplasts are amplified so readily by the universal bacterial primers (515F/806RB), the mismatch between positions 783–799 (based on *E.coli* numbering), just upstream of our 806RB primer, has been targeted by bacterial primers designed to reduce chloroplast contamination of 16S data (Hanshew et al., 2013). Within this region, 15 bases upstream of the V4-806 primer-binding site, we observed two mismatches to the *E.coli* 16S rRNA (GT vs TA) across all the eukaryotic sequences we evaluated. This mismatch could be targeted in designing a diatom-specific 16S primer pair.

Further research is required to validate the performance of 16S primers across diatom taxa. A pairwise test of the V4 regions from 16S and 18S assays on a mock community of prokaryotes and photosynthetic eukaryotes would provide fundamental data on primer bias and efficiency. Needham & Fuhrman (2016) found highly concordant patterns of phytoplankton dynamics when comparing 16S and 18S abundance estimates but their direct comparison is unusual. Most investigations have considered the prokaryotic community using 16S and the eukaryotic community using 18S (e.g., Brinkmann et al., 2015; Laroche et al., 2018; De Sousa et al., 2019). Additional details about the diatom assemblage could be gathered by analysing the discarded 16S plastid sequences from these studies alongside the 18S results. Eiler (2013) suggested 16S as an ideal first step analysis that could be coupled to a second method such as 18S with higher taxonomic resolution and deeper sampling of protist diversity. In cases where 18S may have provided ambiguous results at lower taxonomic levels, 16S reads could supply additional resolution and the diversity. To our knowledge, this has not been tested but the large volume of publicly available 16S microbiome research means that supplemental data may be available locally or regionally.

The use of PhytoREF to assign taxa to the eukaryotic fraction of 16S reads is becoming a common practice (e.g., Zamora-Terol, Novotny & Winder, 2020; Alcamán-Arias et al., 2021) but the taxonomic resolution for PhytoREF is limited, even for marine taxa. In their marine bacterioplankton analysis, Milici (2016) found 59–69% assignment at the order level and only 16–24% at the genus level. Our efforts to classify the 16S Bacillariophyta reads in NCBI against PhytoREF was less specific, with only 33.8% of the sequences assigned an order.

According to the PhytoREF taxonomic assignment of our biofilm chloroplast sequences, the highest proportion (36.4%) were attributed to diatoms. Of the 71 diatom OTUs with at least 1% abundance in a sample, three were identified to genus. These identifications are suspect, however, as only *Navicula* was observed under the microscope and the other two genera are predominantly marine. The *Chaetoceros* genus contains some freshwater species but *Psammodictyon* is considered a marine genus and is therefore unlikely to be encountered (Round, Crawford & Mann, 1990). There were 65 diatoms that could not be classified at the order level which may reflect the limited representation of freshwater microalgae in the PhytoREF library, the limited protist databases (Pawlowski et al., 2016), and the lack of molecular data for Australian diatoms. In light of the ongoing accumulation of sequences and refinements to diatom phylogeny and taxonomy, an updated version of PhytoREF, including freshwater algae, would be a valuable resource.

### Applications of the method

Currently, 16S sequences from freshwater diatoms can be utilised using a taxonomy-free approach (Pawlowski et al., 2016; Apothéloz-Perret-Gentil et al., 2017) that does not rely upon on identification. Under this approach, diatom community data mined from existing 16S reads can be related directly to ecological conditions to help fill knowledge gaps around diatom phylogeny and ecology and to develop novel stream health indices. We suggest that three ‘data clouds’ exist for identifying and classifying diatoms: morphological, molecular, and environmental. Improved information about a morphological species, an OTU (DNA barcode), or a set of environmental variables expands a given ‘data cloud’ and barcodes serve as stable identification benchmarks to link records between ‘clouds’ and through time (Zimmermann et al., 2014). DNA barcodes also serve as indelible fingerprints when taxonomic reshuffling challenges the identity of existing morphospecies (Zimmermann et al., 2014). Under the ‘data cloud’ model, data can accumulate simultaneously to establish biotic and abiotic relationships over time. The ten diatom OTUs that we documented in all four locations from inland, freshwater sites to estuarine seagrass communities (Fig. 3A) are an example of how barcodes can link distant and diverse sites. The value of this information increases, if or when, existing morphological records and site-level environmental conditions are compared across locations.

In this study, the 67 diatom OTUs that were shared between our field samples and public database samples describe the occurrence of similar diatom taxa across diverse habitats at a continental scale (Figs. 1B, 3A, 3B). The limited overlap of 27 OTUs with the nearby estuary site in Victoria (VIC) could reflect environmental differences or there may be selective pressures that restrict the diatom assemblage on seagrass leaves. There is potential to consider this and other ecological questions by assembling diatom community patterns from microbial data that were generated for another purpose. For example, the samples that O’Dea et al. (2019) sequenced to track the microbial signatures of wastewater shared 34% of the diatom OTUs from coastal streams in Victoria (BIOFILM). Our analysis shows high overlap (86%) with the samples that Kaestli et al. (2019) used to compare microbial communities in perennial and ephemeral water bodies in the Australian arid zone (NT), suggesting similar diatom communities among the distant sites. It should be noted that this similarity could be, in part, a product of the larger sample number (*n* = 78) relative to that of the VIC site (*n* = 5). Kaestli et al. (2019) describe a consistently large proportion of cyanobacteria across their samples but our consistent detection of diatom OTUs suggests that the proportion could be skewed due the misattribution of chloroplast sequences in standard 16S rRNA databases.

In this study, we tested whether 16S amplicon reads from stream biofilm samples could describe local diatom assemblage patterns, and then verified the approach on a larger but limited biogeographic scale. However, scaling up this approach to broader diatom biogeographic ranges could be considered by directly mapping public 16S libraries against PhytoREF, as shown in different systems. For example, del Campo et al. (2017) screened 16S sequences to study the global distribution of the green algae, *Ostreobium*, and documented consistent co-occurrence with hard coral. A similar approach could evaluate the degree of community similarity and test assumptions of cosmopolitan diatom distribution and ecological preferences (Gell, 2019).

Accurate, efficient, and cost-effective characterization of diatoms could be widely beneficial across an array of ecological and management applications. For example, several toxic diatom species are monitored in coastal Australian regions due to their role in harmful algal blooms (HAB) (Ajani et al., 2020). HAB surveillance monitoring would benefit from the use of a single amplicon, such as our approach here, that could characterize both diatom and cyanobacteria populations. As established bioindicators, diatom community structure could also augment the assessment of wastewater treatment methods that have so far, relied upon microbial communities (Chonova et al., 2016; Stoeck et al., 2018). Diatom community patterns have even been used to trace the past locations of sea turtles (Rivera et al., 2018) and human bodies (Scott et al., 2014) and digging diatom data out of 16S microbiome studies presents a promising opportunity to advance biosurveillance, forensic, biodiversity, and bioassessment efforts.

## Conclusions

We show that in-depth diatom community data can be uncovered from existing but underutilized 16S rRNA plastidal sequences from microbial community profiling. Even in a poorly studied region, diatom OTUs filtered from 16S chloroplast reads can describe community composition and improved characterization of the chloroplast reads may, in some cases, lead to different conclusions about community dominance and water quality. Digging into existing 16S datasets may inform phylogeny in regions where diatoms have been extensively studied, or may provide a first pass for detecting diatoms and considering broad spatial relationships in regions of limited research. This study provides proof of concept for the mining of digital diatom sequences, a method, which could be applied to local, regional, and global research questions.

We suggest that the universality of the 515/806 primer and the variability of the 16S region warrant further investigation as a tool to characterize photosynthetic eukaryotes. With additional primer performance validation and improved reference databases, the massive volume of publicly available environmental microbiome data could potentially provide a treasure trove for studying diatoms and other microalgae with minimal field or laboratory costs.

##  Supplemental Information

10.7717/peerj.11576/supp-1 Supplemental Information 1 Taxonomic assignment of key OTUs by different databases

10.7717/peerj.11576/supp-2 Supplemental Information 2 Sample reads and OTU taxonomy

10.7717/peerj.11576/supp-3 Supplemental Information 3 Chloroplast reads and 16S rRNA (A) Contribution of each major group to the 16S reads for each site (9 samples per site). (B) Chloroplast reads by phylum ( >1%). (C) Ochrophyta reads by class ( >1%, diatoms as Bacillariophyta). (D) Alignment of the PCR primers to selected 16S rRNA plastid sequences used MAFFT (default setting).

10.7717/peerj.11576/supp-4 Supplemental Information 4 NCBI sequences and PhytoREF Proportion of the publicly available, partial ‘Bacillariophyta’ 16S sequences (1666 total) in NCBI that were classified at each taxonomic level against the PhytoREF database.

10.7717/peerj.11576/supp-5 Supplemental Information 5 Chlorophyll a concentration, calculated by sample and by area Chlorophyll a concentration of each sample is calculated using the concentration of the subsample (0.5 mL) and total sample volume. The concentration per unit area is calculated using the total sampled surface area of 0.018 m ^−2^.

## Additional Information and Declarations

### Field Study Permissions

The following information was supplied relating to field study approvals (i.e., approving body and any reference numbers): Access was approved by the Victoria Department of Environment, Land, Water and Planning (permit # 10008062).

### Data Availability

The following information was supplied regarding data availability: The sequences from biofilm field samples are available at NCBI SRA: BioProject PRJNA588337.
