# Supplementary material for: Dumpster diving for diatom plastid 16S rRNA genes
Source: PeerJ. 2021 Jul 1;9:e11576. doi: 10.7717/peerj.11576 (PMC8255066; doi:10.7717/peerj.11576)
Supplement: Supplemental Information 1 [file peerj-09-11576-s001.pdf]

OTU	NCBI restricted			NCBI default			PhytoRef
	Accession #	Taxonomy	% Id	Accession #	Taxonomy	% Id	Taxonomy
1	KY498709.1	Angulodiscorbis quadrangularis isolate AQ159-89	99%	MH934704.1	Uncultured bacterium clone Sum14ACA187	99%	Kingdom.Eukaryota;Supergroup.Stramenopiles; Phylum.Ochrophyta; Class.Bacillariophyta; Subclass.Bacillariophyta_X; Order.Naviculales; Suborder.Naviculales_X
	KY498706.1	Glabratella patelliformis isolate GP446-17	99%	KT977116.1	Uncultured prokaryote clone	99%	
	KP792485.1	Planoglabratella opercularis isolate GO863-27	99%	KF964592.1	Uncultured bacterium clone Sum14ACA187	99%	
6	KY499654.1	Gomphoneis minuta var. cassieae chloroplast	99%	KM134804.1	Uncultured bacterium clone LNH_9_9_11_Water.252201	99%	Kingdom.Eukaryota; Supergroup.Stramenopiles; Phylum.Ochrophyta; Class.Bacillariophyta; Subclass.Bacillariophyta_X; Order.Chaetocerotales; Suborder.Chaetocerotales_X; Family.Chaetocerotaceae; Genus.Chaetoceros; Species.Chaetoceros
	KC509523.1	Didymosphenia geminata chloroplast	98%	KM133341.1	Uncultured bacterium clone LNH_9_9_11_Water.207325	99%	
	MH011748.1	Chaetoceros sp. isolate GF104-16S_7	97%	KM133339.1	Uncultured bacterium clone LNH_9_9_11_Water.207294	99%	
8	KT952293.1	Pinnularia sp. U-strain	97%	AY212583.1	Uncultured bacterium clone 133ds10	99%	Kingdom.Eukaryota; Supergroup.Stramenopiles; Phylum.Ochrophyta; Class.Bacillariophyta; Subclass.Bacillariophyta_X
	FJ002185.1	Pennate diatom sp. CCAP 1008/1 16S	97%	MG715848.1	Uncultured bacterium clone 293	98%	
	HM449710.1	Navicula minima 12S ribosomal RNA	97%	JF929325.1	Uncultured cyanobacterium clone CMMG12	98%	
30	LN735382.3	Fragilaria sp. RCC2508 chloroplast	99%	KC246081.1	Uncultured cyanobacterium clone XSLA025	99%	Kingdom.Eukaryota; Supergroup.Stramenopiles; Phylum.Ochrophyta; Class.Bacillariophyta
	LN735323.3	Synedropsis sp. RCC2043 chloroplast	99%	JQ654955.1	Uncultured bacterium clone lagoon_D14	99%	
	FJ002235.1	Synedra hyperborea isolate C44 16S	99%	EU290435.1	Uncultured bacterium clone Tc48Tet1mesTet2ect	99%	
104	KT952293.1	Pinnularia sp. U-strain	97%	AY212583.1	Uncultured bacterium clone 133ds10	99%	Kingdom.Eukaryota; Supergroup.Stramenopiles; Phylum.Ochrophyta; Class.Bacillariophyta; Subclass.Bacillariophyta_X
	FJ002185.1	Pennate diatom sp. CCAP 1008/1	97%	MN156759.1	Uncultured bacterium clone A204	97%	
	HM449710.1	Navicula minima 12S ribosomal RNA	97%	JF280491.1	Uncultured bacterium clone GBX-B-COQ1-157	97%	
738	LN735309.2	Psammodictyon sp. RCC1970 chloroplast	99%	KP076635.1	Uncultured bacterium clone M8UC_PoM_110m_20	99%	Kingdom.Eukaryota; Supergroup.Stramenopiles; Phylum.Ochrophyta; Class.Bacillariophyta; Subclass.Bacillariophyta_X; Order.Surirellales; Suborder.Surirellales_X; Family.Surirellaceae; Genus.Psammodictyon; Species.Psammodictyon
	KY498708.1	Glabratella patelliformis isolate GP446-20	99%	JN986365.1	Uncultured bacterium clone U1370-196	99%	
	KY498705.1	Glabratella patelliformis isolate GP446-16	99%	JF272054.1	Uncultured bacterium clone 8M73	99%	
2769	KY498709.1	Angulodiscorbis quadrangularis isolate AQ159-89	100%	KY498709.1	Angulodiscorbis quadrangularis isolate AQ159-89	100%	Kingdom.Eukaryota; Supergroup.Stramenopiles; Phylum.Ochrophyta; Class.Bacillariophyta; Subclass.Bacillariophyta_X; Order.Naviculales; Suborder.Naviculales_X; Family.Naviculaceae; Genus.Navicula; Species.Navicula
	KY498706.1	Glabratella patelliformis isolate GP446-17	100%	KY498706.1	Glabratella patelliformis isolate GP446-17	100%	
	KP792485.1	Planoglabratella opercularis isolate GO863-27	100%	MF361026.1	Uncultured bacterium clone C11	100%	
4485	AF277540.1	Diatom sp. ARCTIC.149	98%	MF451026.1	Uncultured bacterium clone OTU1470	99%	Kingdom.Eukaryota; Supergroup.Stramenopiles; Phylum.Ochrophyta; Class.Bacillariophyta; Subclass.Bacillariophyta_X
	AF277476.1	Diatom sp. SIC.42333	98%	MH819045.1	Uncultured diatom clone H1910-16S_42	99%	
	MK045450.1	Halamphora americana chloroplast	98%	MH819039.1	Uncultured diatom clone H1910-16S_35	99%	
4668	LN735393.3	Bacillaria paxillifer chloroplast	98%	FJ355395.1	Uncultured organism clone 051011_S3_142	99%	Kingdom.Eukaryota; Supergroup.Stramenopiles; Phylum.Ochrophyta; Class.Bacillariophyta; Subclass.Bacillariophyta_X
	FJ002242.1	Leyanella arenaria isolate C70	98%	MH818908.1	Uncultured diatom clone H1724-16S_07	98%	
	FJ002223.1	Cylindrotheca closterium isolate C16	98%	KJ811885.1	Uncultured bacterium clone 0727N7_3_2_F09838	98%	
11469	FJ002185.1	Pennate diatom sp. CCAP 1008/1	98%	MN156759.1	Uncultured bacterium clone A204	98%	Kingdom.Eukaryota; Supergroup.Stramenopiles; Phylum.Ochrophyta; Class.Bacillariophyta; Subclass.Bacillariophyta_X
	MK045450.1	Halamphora americana chloroplast	98%	MF558965.1	Uncultured bacterium clone 3383	98%	
	NC_037997.1	1 Plagiogrammopsis vanheurckii chloroplast	98%	KT013522.1	Uncultured prokaryote clone OTU_214	98%	
21053	JQ088178.1	Synedra acus chloroplast	99%	LC065762.1	Uncultured bacterium gene	99%	Kingdom.Eukaryota; Supergroup.Stramenopiles; Phylum.Ochrophyta; Class.Bacillariophyta
	KP792487.1	Bacillariophyta sp. 867-32	98%	LC065737.1	Uncultured bacterium gene	99%	
	KP792478.1	Planoglabratella opercularis isolate GO_m25-21	98%	LC065719.1	Uncultured bacterium gene	99%	
