# Supplementary figures and images for: Dumpster diving for diatom plastid 16S rRNA genes
Source: PeerJ. 2021 Jul 1;9:e11576. doi: 10.7717/peerj.11576 (PMC8255066; doi:10.7717/peerj.11576)

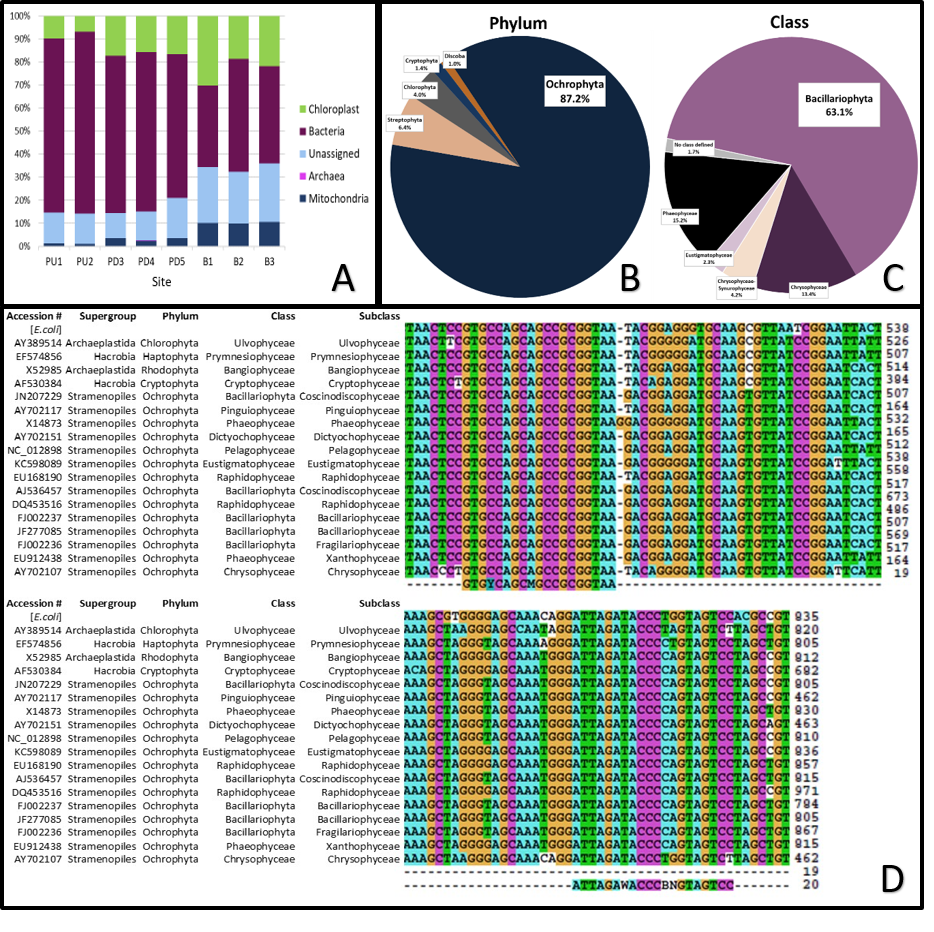

Supplement: Supplemental Information 3 — (A) Contribution of each major group to the 16S reads for each site (9 samples per site). (B) Chloroplast reads by phylum ( >1%). (C) Ochrophyta reads by class ( >1%, diatoms as Bacillariophyta). (D) Alignment of the PCR primers to selected 16S rRNA plastid sequences used MAFFT (default setting). [file peerj-09-11576-s003.png]

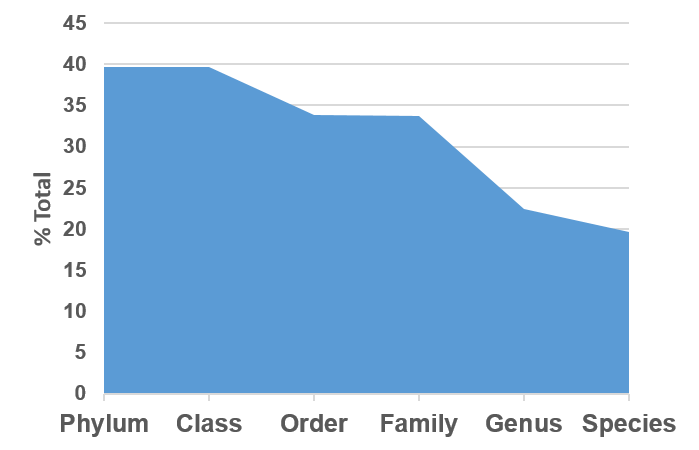

Supplement: Supplemental Information 4 — Proportion of the publicly available, partial ‘Bacillariophyta’ 16S sequences (1666 total) in NCBI that were classified at each taxonomic level against the PhytoREF database. [file peerj-09-11576-s004.png]
